# Adjusting and doing the same: school nurses’ descriptions of promoting participation in health visits with children of foreign origin

**DOI:** 10.1186/s12889-020-10144-2

**Published:** 2021-01-09

**Authors:** Emmie Wahlström, Marie Golsäter, Mats Granlund, Inger K. Holmström, Peter Larm, Maria Harder

**Affiliations:** 1 ChiP research group, School of Health, Care and Social Welfare, Mälardalen University, Box 883, 721 23 Västerås, Sweden; 2 School of health, care and social welfare, Mälardalen University, Västerås, Sweden; 3 CHILD-research group, School of Health and Welfare, Jönköping University, Jönköping, Sweden; 4 Child Health Services and Futurum, Region Jönköping County, Jönköping, Sweden; 5 Department of Public Health and Caring Sciences, Uppsala University, Uppsala, Sweden; 6 Child Health Care Services, Region Västmanland, Västerås, Sweden

**Keywords:** School health services, School nursing, Children, Cultural diversity, Participation, Content analysis

## Abstract

**Background:**

School nurses in the school health services are assigned to promote health and participation among children when conducting health visits. Still, for children of foreign origin this promotion of participation might be hampered by challenges related to cultural diversity and language barriers. Therefore, knowledge needs to be developed regarding how these children’s participation can be promoted, to support them in sharing and describing matters important for their health. The aim was to investigate school nurses’ descriptions of promoting participation for children of foreign origin in health visits.

**Methods:**

A content analysis of 673 Swedish school nurses’ answers to eight open-ended questions regarding promotion of participation for children of foreign origin was conducted. The open-ended questions were part of a larger web-based cross-sectional survey distributed to school nurses in Sweden.

**Results:**

The results show that school nurses use three main approaches during the health visit: adjusting according to the child’s proficiency in Swedish and/or cultural or national background, adjusting according to the child’s individual needs, and doing the same for all children regardless of their origin. Yet, adjustments according to the child’s proficiency in Swedish and/or cultural or national background were the most common.

**Conclusions:**

By combining the approaches of adjusting, a child-centered care that contributes to children’s participation in health visits and equity in health could be provided.

## Background

Children’s right to participation in all health care services is regulated by the Swedish patient act [1] and recognized in the Convention on the Rights of the Child [2, 3]. These documents state that children should be heard in matters that concern them [2], that they should be informed about the care provided to them, and that their needs and wishes should be considered [1].

The school health services in Sweden are assigned to promote health and participation among children by the school nurses conducting health visits. These visits are free of charge and provided regularly to all children at the ages of 6, 10, 13–14 and 16 years old [4]. The health visits consist of a health dialogue and physical examinations conducted by a school nurse to promote children’s health and development and to detect risks of ill-health. The health dialogue focus on the child’s development toward an independent life [4–6]. This is done by elaborating on the children’s resources in their daily living [7] and identifying risks of ill-health. The physical examinations focus on height, weight, eyesight, hearing and the spine [4]. Hence, how school nurses act to promote participation for children within the school health services is important for enforcing their legal rights. Although, school nurses adjust their actions according to the child’s age, maturity and experience to promote participation [4] little is known about their promotion of participation when encountering children of foreign origin. Children of foreign origin, i.e. those born abroad or having two parents both born abroad [8], currently represent about 35% of the children in Sweden [9]. This heterogeneous group have an accumulation of health-related risk factors related to migration [10–16] which might be exacerbated by them not being able to participate on their own terms. Furthermore, if actions of promoting participation for children of foreign origin are based on knowledge regarding promoting participation for the majority population of children, the needs of children of foreign origin might not be fully addressed.

In previous research regarding encounters between children of foreign origin and school nurses, both children and school nurses report challenges [16–18]. School-aged children of foreign origin in other care contexts report challenges due to their sometimes-limited language proficiency, lack of trust in the health care services [11, 19] as well as lack of knowledge about the health care services [19]. School nurses experience challenges related to lack of knowledge about trauma-informed care and intercultural nursing as well as to self-awareness [20]. They also report difficulties when addressing specific health-related topics requiring adaptation of content in national guidelines to the child’s culture or origin [17]. Additional challenges are risks of ethnocentricity and stereotyping [21].

Regardless of these challenges, children have the right to participate in health visits on their own terms. For this study, participation is defined as taking part or actively contributing to a situation, event, process or outcome [22]. This includes a combination of attendance (i.e. being in a situation) and involvement (i.e. subjective experience of engagement and motivation) [23]. Yet, participation is not merely considered an action or state, but also an interactional position [24] influenced by the interaction or transaction between the individual, the surrounding social context and the social environment [23, 25].

School-aged children, regardless of origin, emphasize the importance of having a possibility to participate and feel respected in health visits [6]. To participate, children need to understand the purpose of the health visit and to know what the school nurse can assist with [26]. Such knowledge will help them to trust that nurses will respect their confidentiality [26, 27] and to express their own view [27, 28]. As there is an asymmetrical distribution of power, where the school nurses are in charge of the agenda of the health visit, the school nurses’ actions of informing the child about the situation and inviting the child to participate is especially important when promoting children’s participation [28]. These actions include school nurses actively listening, creating a pleasant caring atmosphere, providing encouragement and respecting the child’s rights [27]. The nurses, when observed, are also found to use similar interaction strategies to promote children’s activity and participation in encounters with children [5]. In addition, related strategies are mentioned as important when encountering unaccompanied refugee children to build trust between these children and health professionals. Besides having a positive attitude, and being open to and showing acceptance of what the child expresses in the dialogue [20], the strategies also include listening to the child before assessing the child’s needs and readiness to be provided with health education or guidance [29].

Yet for children of foreign origin, the importance of participation is sparsely investigated in previous research. As children’s participation is a fundamental right [1–3], and cultural norms and ideals of participation integrated in a child’s beliefs might conflict with those in the school health services [30] further knowledge regarding the participation of children of foreign origin in health visits is needed. Therefore, studying descriptions of school nurses’ promotion of participation for children of foreign origin in health visits may provide further insights into their actual practice.

### Aim

The aim is to investigate school nurses’ descriptions of promoting participation for children of foreign origin in health visits.

## Method

### Study design

This study was a descriptive qualitative study [31] analyzing answers to open-ended questions extracted from a larger cross-sectional web-based survey of school nurses in Sweden. The survey consisted of a questionnaire containing 49 items focusing on three areas: cultural competence, promotion of participation, and demographic items. For this study, the analysis focused on answers to the open-ended questions concerning promotion of participation (*n* = 673). The items on cultural competence are reported elsewhere [32].

### Setting and participants

The study population consisted of all school nurses in Sweden (N~ 4000). School nurses in Sweden follow the national guidelines for the school health services [4], but the local organization of the services provided might vary. Still, all children should have access to a school nurse. As there is no national record of all school nurses working in Sweden, e-mail addresses to every school nurse, working in both municipal and private schools, were identified manually. To identify school nurses’ e-mail addresses, municipal officials and administrators at private schools in Sweden’s 290 municipalities were contacted. Schools in 270 municipalities agreed to participate. No exclusion criteria were applied. In total, 3331 e-mail addresses to school nurses were identified.

### Data collection

An e-mail containing information about the study and a link to the web-based questionnaire was sent to school nurses during August 2017 to January 2018 (*N* = 3331). After one week a reminder was sent, followed by a second reminder after one additional week. The survey closed two weeks after the last reminder. The questionnaire was completed by 816 respondents (24%) and the e-mail was opened by 1233 nurses (37%). The software (EsMaker) used enabled respondents to choose whether or not to provide an answer to the open-ended questions. All respondents that provided at least one answer to a question on promotion of participation were included in the analysis (*n* = 673). Respondents answering “not relevant” or similar to all questions were excluded from the analysis. Examples of irrelevant answers were: “not relevant”, “only have children of Swedish origin at my school”, “do not encounter children of foreign origin”, “no experience”.

School nurses’ promotion of children’s participation in health visits with children of foreign origin was investigated using eight open-ended study-specific questions, see Table 1. These questions followed after 23 questions about cultural competence. The content and phrasing of the questions were developed from the results of previous studies of nurses’ promotion of children’s participation in child and school health services [28, 33] and aspects of learning, communication and interaction in ICF-CY [34]. The questions were tested for face-validity using the think-aloud technique, which resulted in minor changes of the wording.

**Table 1:** The open-ended questions on promotion of participation

Question no.	Open-ended question
24	To what extent do you inform the child about the purpose of the health visit and how it will be conducted according to the guidelines of the school health services?
25	Before the health visit with children and family of foreign origin, how do you prepare yourself?
26	How do you form/design the health visit?
27	How do you develop trust with children and families?
28	How do you meet the children’s expectations, wishes and needs?
29	How do you show that you are paying attention to the child?
30	How do you show the child that you are listening?
31	When you encounter children of foreign origin, how do you communicate in other ways than verbally?

The demographic items concerned the school nurses’ age, sex, country of origin, spoken language, educational level, years working in school health services and frequency of encountering children of foreign origin (assessed on a Likert scale). These were used to describe the characteristics of the respondents to the open-ended questions, see Table 2.

**Table 2:** Characteristics of respondents

Characteristic	n	(%)
Sex (*n* = 673)		
Female	664	98.7
Male	7	1.0
Other / will not report	2	0.3
Age (*n* = 665; Mean = 50.3 (SD: 9.1))		
< 40 years	105	15.8
41–50 years	214	32.2
51–60 years	259	38.9
61 < years	87	13.1
Clinical experience in SHC (*n* = 673; Mean = 8.4 (SD: 7.3))		
0–5 years	306	45.5
6–10 years	145	21.5
11–15 years	106	15.8
16 < years	116	17.2
Country of origin (*n* = 671)		
Sweden	632	94.2
Nordic country (Sweden excluded)	16	2.4
European country (Nordic countries excluded)	14	2.1
Country outside Europe	9	1.3
Spoken languages (*n* = 669)		
Swedish	59	8.8
One additional language	350	52.3
Two or more additional languages	260	38.9
Specialist education (*n* = 666)		
Public health nursing^a^	339	50.9
Pediatric nursing^a^	180	27.0
School nursing^a^	65	9.8
Public health and pediatric nursing^a^	24	3.6
Other specialist education	34	5.1
No specialist education	24	3.6
Frequency of encountering children of foreign origin (*n* = 673)		
Once or several times a day	282	41.9
Once or several times a week	189	28.1
Once or several times a month	131	19.5
Less than once a month	71	10.5

^a^Education including training in child or adolescent nursing

### Data analysis

**Fig. 1:**
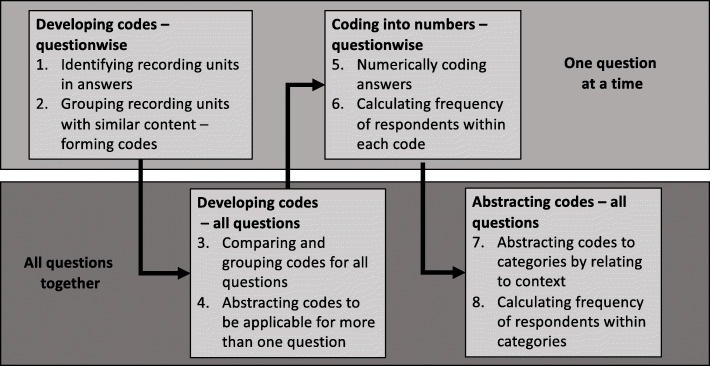
Overview of the analysis process based on Krippendorff [35]

Answers to the open-ended questions were analyzed using content analysis according to Krippendorff [35], see Fig. 1. In total, the eight open-ended questions provided 5269 answers by 673 school nurses. All answers were included in the analysis.

Initially each question was analyzed separately, meaning that each answer was read through and related to the specific question (no. 1 in Fig. 1). This was done as the answers did not always contain text describing the context. For example, an answer to the question about information (no. 24, Table 1) was: “start off by briefly describing what we are going to do and why. Highlight that the child is in focus”. When reading through the answers, recording units were also identified one question at a time. A recording unit is defined as a word or phrase expressing a variation in the answer to the question [35]. Each unique answer could include several recording units due to variations of words or phrases in the answers. This can be exemplified by using the quotation above, which includes three recording units: “start off by”, “describing what we are going to do and why” and “highlight that the child is in focus”. Recording units containing similar content were grouped, and this process generated codes specific for each question (no. 2 in Fig. 1). These codes were collated and abstracted to question-specific sub-categories. When the answers to all questions had been worked through (no. 1 and 2 in Fig. 1), the codes and sub-categories for all questions were compared and revised to make sub-categories and codes applicable for more than one question (no. 3 and 4 in Fig. 1). The coherence of codes and sub-categories was discussed and agreed upon between authors (EW & MH).

All codes were then assigned a numerical code. Using these, all recording units were read through once again and provided with a numerical code (no. 5 in Fig. 1). During this process the links among respondent, answer and recording unit were kept, making it possible to count how many respondents had reported an answer containing a certain code. Numerical coding was mainly performed by the first author (EW) but seconded by MH for recording units that evoked uncertainty. When numerical coding was completed, the frequencies of responses for each code was calculated and content in sub-categories was summarized (no. 6 in Fig. 1). By discussing the summaries and relating these to the context of health visits with children of foreign origin, the sub-categories were revised and abstracted to three categories describing the promoting of participation in the health visit (no. 7 in Fig. 1). The categories describing promoting participation in health visits with children of foreign origin included *to adjust the preparations for the health visit*, *to conduct health visits adjusted to the child* and *to follow up on the child’s health-related needs after the health visit.* In addition, the answers contained different descriptions of adjustments and non-adjustments related to the child that were considered when promoting the participation of these children. These aspects were abstracted to a category that should be considered as embedded in the process of the health visit. This category describes school nurses using varying *approaches to health visits with children of foreign origin*. All authors contributed to the discussions. Lastly, the frequencies of respondents within each category and main category were calculated (no. 8 in Fig. 1).

## Results

**Fig. 2:**
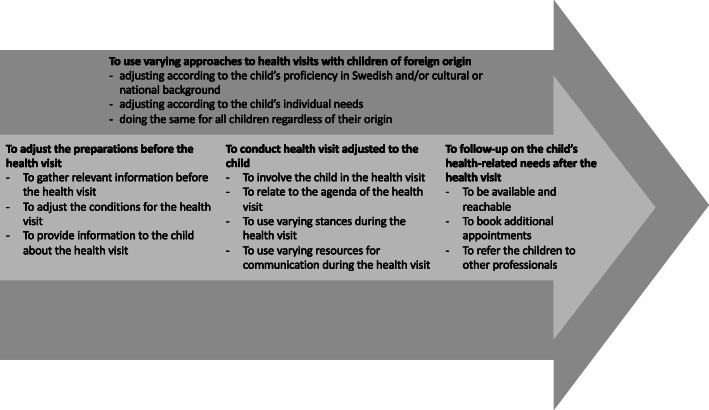
Overview of the categories and sub-categories

The school nurses’ descriptions of promotion of participation for children of foreign origin in health visits show that the school nurses *use varying approaches to the health visit*, see Fig. 2. These approaches are embedded in the process of promoting children’s participation, and they guide the school nurses’ choice of actions and views on the child’s needs when *adjusting preparations before the health visit*, *conducting the health visit adjusted to the child* and *following up on the child’s health-related needs after the health visit*. In the following section the number and percentage of school nurses whose answers incorporated a category, sub-category or code is presented in brackets. These quantities are presented as an indication of how frequently something was mentioned, in order to highlight the variation in the results.

### To use varying approaches to health visits with children of foreign origin

School nurses (SNs) use varying approaches to promote participation in health visits when preparing, conducting and following up on health visits with children of foreign origin. These approaches are: *adjusting according to the child’s proficiency in Swedish and/or cultural or national background* (616 SNs, 92%); *adjusting according to the child’s individual needs* (240 SNs, 36%); or *doing the same for all children regardless of their origin* (307 SNs, 46%). Although these are separate approaches, school nurses describe using one or several of them in a health visit. However, *adjustments according to the child’s proficiency in Swedish and/or cultural or national background* was the most common approach used. By *adjusting according to the child’s proficiency in Swedish and/or cultural or national background* school nurses simplify their way of talking by using easier wording and providing more explanations. They also describe considering cultural aspects when asking questions, as well as asking questions about the child’s background or culture and about specific health topics such as gender mutilation. When school nurses *adjust according to the child’s individual needs* they describe adjusting the content of the health visit to the child’s interests, wishes, expectations and needs as well as their maturity, age and abilities. They do so by focusing on what the child expresses in the health visit, and emphasize the need to encounter each child as a unique individual. In the answers, the school nurses also describe *doing the same for all children regardless of their origin.* This is described as doing the same for children of foreign origin as for children of Swedish origin or not doing anything particular for children of foreign origin. When doing the same the school nurses also stated that they did not give special treatment based on origin or ethnicity.

### To adjust the preparations before health visits

The school nurses describe adjusting the preparations before health visits with children of foreign origin as: *gathering relevant information before the health visit*; *adjusting the conditions for the health visit*; and *providing information to the child about the health visit*.

School nurses describe that the *information they gather before the health visit* mainly focuses on the child’s proficiency in Swedish and native language (286 SNs, 43%), and on the cultural or national background (174 SNs, 26%). They also gather information on health-related data and living conditions for each child. Information is gathered by reading previous documentation, records or health surveys (150 SNs, 22%) and talking to other professionals that encounter the child in school (87 SNs, 13%). Besides information about the specific child, some of the school nurses also gather information on specific topics such as vaccination rates, female gender mutilation and prevalence of tuberculosis (50 SNs, 7%).

Furthermore, the school nurses prepare by *adjusting the conditions for the health visits*. Most of them adjust the conditions by engaging an interpreter (365 SNs, 54%), and by extending the appointment (110 SNs, 16%). Extending the appointment might be a prerequisite for communicating through an interpreter. However, the school nurses also state that they extend the appointment to be able to explain in detail and avoid misunderstandings. In addition, they describe spending more time on confirming appointments with children of foreign origin than with other children. Another adjustment is to arrange the seating and the materials to be used. The adjustments are made as siblings can be present when care-givers are invited, but also to make the child comfortable.

The school nurses also prepare by *providing information to the children* about the purpose of the health visit (268 SNs, 40%). This information is mainly provided in writing, before the visit to a group of children. It predominantly describes “the what and the how” of the health visit (235 SNs, 35%), by explaining the content of the health visit and procedures included. A few school nurses describe the organization of the school health services and their profession (26 SNs, 4%), while other explain that the visits are voluntary, are confidential and that all children are invited to attend these visits (35 SNs, 5%).

Although there are different aspects of preparations, most school nurses (644 SNs, 96%) describe preparing for the health visits with children of foreign origin. Some school nurses even state that they prepare themselves more for health visits with children of foreign origin (96 SNs, 14%) or that they have certain routines for their preparations (29 SNs, 4%). However, a small proportion of school nurses (22 SNs, 3%) state that they do not have to prepare or that they rely on their previous experience.

### To conduct health visits adjusted to the child

School nurses describe conducting health visits adjusted to the child as: *involving the child in the health visit*; *relating to the agenda of the health visit; using varying stances during the health visit* and *using varying resources for communication during the health visit*.

Most school nurses describe *involving the child in the health visit* as engaging in a dialogue by: listening to and looking at the child (536 SNs, 80%); asking questions and asking the child to describe their wishes and needs; summarizing and recapping what the child is describing; and showing respect and providing space for the child. Some school nurses also emphasize using their body to acknowledge the child, by putting a hand on a shoulder or giving a hug (64 SNs, 10%). Furthermore, they describe aspects of involvement such as promoting the child’s resources and skills (178 SNs, 26%), creating a relationship and together with the child shape the health visit to meet the child’s health-related needs.

When conducting the health visits, the school nurses also *relate to the agenda of the health visit* by once again explaining the purpose and content of the health visit (374 SNs, 56%). In addition, they examine, assess and address the child’s health-related needs (262 SNs, 39%). Some school nurses state that they use a predetermined structure (86 SNs, 13%) or have certain preferences (108 SNs, 16%) for the entire health visit. These preferences concern having the health dialogue before or after the physical examinations as well as ending the health visit with the child’s questions.

In addition, the school nurses describe *using different stances during the health visit*. The most frequently described stance is being present in the dialogue (256 SNs, 38%), which implies paying attention and being receptive. Other described stances are being: genuine (5 SNs, 1%); kind and pleasant (127 SNs, 19%); factual and calm (89 SNs, 13%); professional (61 SNs, 9%); helpful and empathic (108 SNs, 16%); as well as being flexible (13 SNs, 2%).

Another aspect of conducting health visits adjusted to the child is *using varying resources for communication* where most school nurses report engaging an interpreter (512 SNs, 76%). The interpreter is mainly professional, but informal interpreters are also engaged, such as friends, family and other school personnel. The interpreter is engaged either for all encounters or when the school nurse believes it is necessary. The school nurses also use other resources to facilitate communication, mainly hand signs, body language and facial expressions (544 SNs, 81%). Other facilitators are: visual resources (images, video, drawing, iPads, 381 SNs, 57%); written or text-based resources (simplified or translated leaflets, simplified health surveys, free online translating programs or web pages, 171 SNs, 25%); and interactional resources (playing together, role play 57 SNs, 8%).

### To follow up on the child’s health-related needs after the health visit

School nurses describe following up on the child’s health-related needs after the health visit as *being available and reachable*, *booking additional appointments* and *referring the children to other professionals*.

To *be available and reachable* is the most frequently described way of following up on the children’s health-related needs (133 SNs, 20%). However, this is not only a strategy to follow up identified needs, but also a way of identifying new needs. The school nurses describe being available and reachable as having an open door, hanging out in the hallways, attending school lunches, quickly replying to messages and greeting the children by name.

If, during the health visit, the school nurses identify needs that warrant further dialogue they *book additional appointments* with the children (86 SNs, 13%). Such appointments are also booked when the time allocated for the original appointment was not long enough or they need to get back to the child regarding specific issues. However, when the school nurses identify needs that they cannot help the children with they *refer the children to other professionals* in the school health team, in other health care services or in local authorities (89 SNs, 13%).

School nurses also describe that they act according to their professional assignment to follow up on the children’s health-related needs and support their health (110 SNs, 16%). A few school nurses (five SNs, 1%) describe efforts extending beyond their professional assignment. These five school nurses apply for financial support from non-governmental organizations for children, contact sports clubs to get the children involved and arrange local fundraisers for clothing for the children.

## Discussion

The school nurses’ descriptions of promoting participation for children of foreign origin in health visits show that they use three main approaches: *adjusting according to the child’s proficiency in Swedish and/or cultural or national background*, *adjusting according to the child’s individual needs*, and *doing the same for all children regardless of their origin*. In a health visit, school nurses may use more than one approach, implying that they balance their usage of approaches in relation to the process of the health visit and the child. The use of these approaches could be interpreted in relation to perspectives incorporated in the guidelines and regulations [4, 36] that school nurses are expected to adhere to, especially perspectives regarding equity in health as well as regarding care based on and sensitive to the needs of the individual child. To further elaborate on the results, these two perspectives will be used to interpret the presented approaches in relation to the context and practice of the school nurse when promoting participation for children in health visits.

### Promoting participation as promoting equity in health

The school nurses’ frequent use of the approach *adjusting according to the child’s proficiency in Swedish and/or cultural or national background* when promoting participation could be interpreted as the school nurses promote equity in health. Thus, implying that they promote equity in health by establishing communication and reducing the influence of the language and cultural barriers described in previous studies [11, 16–19]. Reducing language barriers is described as decreasing inequalities in health in the population [37]. By reducing language barriers, the children might also feel included and might better understand the information provided by the school nurse. These two factors are related to participation in health care from the perspective of children [38] and could be understood as necessary for the children to actively contribute to the situation [22]. Still, being able to communicate in a shared language might not be sufficient in regards to the children understanding and interpreting what is being said related to health in the encounter [39]. Being able to understand information about health requires health literacy. However, findings show levels of health literacy among a majority of adult refugees being inadequate or limited [40] and research on the health literacy of children of foreign origin seem to be sparse, as no studies with in the EU regarding these children’s health literacy was found in a recent systematic review [41]. This lack of research and the possibility of health literacy influencing children’s participation in health visits warrant further studies regarding the health literacy of children of foreign origin and implications for both their participation in health visits and equity in health.

The school nurses being available and reachable when following up on children’s health-related needs is also described as promoting participation for children of foreign origin. By being available and reachable the school nurse provide access to the health visits, which is a prerequisite for participation [23]. Access to health care is also vital in promoting equity in health [42]. Still, being a child of foreign origin is related to having a higher risk of experiencing difficulties in accessing a school nurse [43], and they also report difficulties in keeping appointments [11]. However, that the school nurses in this study describe availability as important for children of foreign origin suggests that these school nurses might be aware of the impact that availability and access have on these children’s participation in health visits and also on promoting equity in health.

By *adjusting according to the child’s proficiency in Swedish and/or cultural or national background* it could further be argued that the school nurses consider the influence of cultural norms on a person’s motivations and choices with regard to participation [30]. Hence, acknowledging the influences of the surrounding social context and the social environment on the child’s participation in the health visit [23, 25]. This approach is supported by conclusions regarding school nurses’ need for cultural knowledge and skills when working with unaccompanied refugee children [20]. It also calls for cultural competence in encounters with children of foreign origin [16, 20, 44]. In addition, the approach might also imply that school nurses consider the migrated person’s expectations when participating in encounters with health care professionals [19]. This approach could be indicative of school nurses using cultural competence as described by Campinha-Bacote [45]; striving to work within the cultural context of the client. However, if *adjusting according to the child’s proficiency in Swedish and/or cultural or national background* is applied without considering the heterogeneity within this group of children and the characteristics of the individual child, it could be argued that the school nurses rather enforce stereotypes and ethnocentricity [46]. It could also be argued that such adjustments might not fully consider the multiplicity of definitions of health nor the cultural or contextual elements of understanding health behaviors [47]. Lack of such consideration might also lead to the children’s own personal, social and cultural identity becoming undermined and might reduce the children’s possibility of participating in the health visit. These aspects raise questions regarding whether *adjusting according to the child’s proficiency in Swedish and/or cultural or national background* throughout the process of the health visit really helps to promote the children’s participation and equity in health.

### Promoting participation as providing child-centered care

The approach of *adjusting according to the child’s individual needs* concerns promotion of participation based on the child’s individual needs and preferences and could be understood as providing child-centered care. Such care includes recognizing individual needs and wishes by using situated skills and strategies while showing the child respect [48]. Child centered care also implies supporting the autonomy of the individual child while considering the cultural and social context of caring. However, the skills and strategies described by the school nurses to promote children’s participation are not unique for this group of children. Previous findings also show nurses balancing the required assignments in the agenda of the health visit with facilitating children’s involvement based on the needs, motivation and interest of the specific child [28]. Furthermore, some of the school nurses’ specific actions of explaining the purpose and content of the health visit are also described by children, regardless of origin, as promoting their participation in the health care [27]. Still, the challenges present in encounters between school nurses and children of foreign origin [18, 20] might indicate that the cultural and social context of children of foreign origin needs to be further considered in order to promote their participation. Further research is warranted to clarify how school nurses can promote participation while considering the cultural and social context of children of foreign origin, as not to contribute to the discourse of color-blindness in nursing inquiry [49].

### Promoting participation as doing both …

Based on the discussion, both approaches of *adjusting according to the child’s proficiency in Swedish and/or cultural or national background* and *adjusting according to the child’s individual needs* are warranted when promoting children’s participation in health visits with children of foreign origin. Still, the results show that 46% of the school nurses (307 SNs) use the approach of *doing the same for all children regardless of their origin* at least once during the process of the health visit. The frequent use of this approach might imply that school nurses do not consider adjustments to be necessary throughout the process of the health visit. Yet, not considering children’s cultural or national background in the process might increase the likelihood of the school nurses overlooking risks of ill-health [50], might hinder the child’s participation [46] and might lead to further inequities in health care [51]. In addition, using the approach of *doing the same for all children regardless of their origin* could be interpreted as ignoring the structural inequalities contributing to differences in health and experiences of health and health care [49]. This approach might also indicate school nurses lacking awareness of implications of not recognizing the heterogeneity within any group of children and especially for this group of children. Such implications might be ethnocentrism, underlying bias or racism and the belittling of these children’s experiences. By using both approaches of adjustment during the process of health visits, challenges known in advance (such as language barriers) could be acknowledged and reduced, and the health visit could be guided by the child’s needs and wishes. These adjustments are related to school nurses recognizing children’s culture and language proficiency as social determinants of health impacting the health outcomes of children [50]. Social determinants of health also need to be considered and explored in health visits since the social environment and social context influence how children become involved and actively contribute in the health visit [23]. In addition, the possibilities and rights of children of foreign origin to share their own experiences, needs and preferences need to be promoted [52]. By adjusting the promotion of participation to the needs of the individual child, based on both individual preferences and the social determinants of health, the participation of children of foreign origin can be promoted in a way that contributes to equity in health.

### Limitations

All eight open-ended questions addressed school nurses’ promotion of children’s participation in health visits with children of foreign origin, although this was only explicitly stated in two questions. By not including the phrase “children of foreign origin” in all questions, some school nurses might have described their promotion of participation for children in general. Still, this risk might be quite small since adjustments in relation to cultural and or national background were common in all categories and the open-ended questions followed a statement declaring that the eight questions concerned children of foreign origin.

In self-reported data there is a risk of social desirability bias [53, 54]. Yet, the consistency of the answers among the school nurses shows that their descriptions are quite similar. This implies that they all either describe the same ideal of health visits and promotion of participation for children of foreign origin or that there are similarities in how the school nurses actually describe their work. Still, the school nurses answering the survey might not fully represent the population of school nurses in Sweden as the effort of answering might indicate that only those with an interest in the topic answered. This might also be related to the limited response rate of 20% for the open-ended questions. However, the sample of school nurses answering the survey consists of school nurses from all counties in Sweden and the demographics of the respondents in this study correspond to demographics of school nurses in Sweden. This might also indicate that the results could be transferred to similar groups of school nurses [55].

Only the manifest content in the answers has been coded in order to avoid interpretation at an early stage in the analysis and to construct coding lists consistent with the quality criteria of Krippendorff [35]. To prevent inconsistency of coding, one author (EW) has coded the data. Discussions about the coding process have been held continuously with another author (MH). EW and MH have also coded about 5–10% of answers for each question together to check consistency and discuss the relevance of the established codes.

Since these results are based on answers to open-ended questions in a survey, the depth of the answers is limited. Still, the volume of answers provides variety in how the school nurses describe promotion of participation. In addition, the categories presented in the result cover the entire process of a health visit although the open-ended questions focused on aspects of preparing for the health visit and conducting the health visit. The follow-up category emanated from data, although no questions specifically addressed this topic.

### Clinical implications

This study provides knowledge about how school nurses describe their promotion of participation among children with an accumulation of risk factors. These findings can be used in discussions in school health services to enhance awareness about adjustments when encountering children of foreign origin. School nurses need to continuously reflect on the adjustments used and how these will contribute to the children’s participation and equity in health. Such reflections include considering social determinants of health when preparing for, conducting and following up on the health visit [50].

## Conclusion

School nurses promote participation for children of foreign origin by using varying approaches to health visits in the process of adjusting their preparations for the health visits, conducting health visits adjusted to the child, and following up on the child’s health needs after the health visit. These approaches guide the school nurse’s choice of actions and views on the child’s needs when promoting participation. Although *adjusting according to the child’s proficiency in Swedish and/or cultural or national background* was the most used approach throughout the process of the health visits, this approach might need to be combined with *adjusting according to the child’s individual needs* to provide a child-centered care that contributes to children’s participation and equity in health. Still, further research is needed regarding the balancing act of using these approaches when encountering and promoting participation of children of foreign origin while considering the influencing structural inequalities and the cultural and social context of each individual child.

## Data Availability

The datasets generated and analyzed during the current study are not publicly available due to privacy and ethical restrictions but are available from the corresponding author on reasonable request.

## Abbreviations

ICF-CY: The International Classification of Functioning, Disability and Health for Children and Youth
SNs: School nurses
